# Behavior change intervention targeting physical activity and diet improves stress and sleep

**DOI:** 10.1371/journal.pone.0343397

**Published:** 2026-03-02

**Authors:** Samuel L. Battalio, Bonnie Spring, Elizabeth Wilson, Donald Hedeker, Angela Fidler Pfammatter

**Affiliations:** 1 Department of Preventive Medicine, Northwestern University Feinberg School of Medicine, Chicago, Illinois, USA,; 2 Department of Behavioral Sciences and Social Medicine, Florida State University College of Medicine, Tallahassee, FL, USA,; 3 Rush Medical College, Rush University, Chicago, IL, USA,; 4 Department of Public Health Sciences, University of Chicago, Chicago, IL, USA,; 5 College of Education, Health, and Human Sciences, Department of Public Health, University of Tennessee, Knoxville, Knoxville, TN, USA; Japanese Academy of Health and Practice, JAPAN

## Abstract

**Background:**

Poor diet, low physical activity, high stress, and poor sleep are prevalent modifiable risk factors for chronic diseases. Synergistic effects of interventions targeting diet and activity on other health risk behaviors such as stress and sleep are understudied.

**Purpose:**

To conduct a secondary data analysis to investigate whether interventions targeting diet and activity produce collateral improvements in stress and sleep.

**Methods:**

Make Better Choices 2 was a randomized clinical trial to test a technology-assisted coaching intervention with modest incentives to improve diet and activity, as compared to a matched intervention targeting improved stress and sleep. Participants (n = 212) were adults (76.4% female, 59% non-white minority, mean age = 40.8 years) with multiple diet and activity risk behaviors. For 7 days at baseline, 3-, 6-, and 9-months, participants reported perceived sleep duration, stress, diet, and activity through a smartphone application. Outcomes were evaluated by linear mixed models. The study was registered on clinicaltrials.gov (NCT01249989)*.*

**Results:**

Both interventions produced significant, statistically comparable improvements in average daily stress rating (z = 1.35, p = .177). Reduction in average daily stress rating was 1.68 following diet/activity intervention (z = −12.25, *P* < .001) and 2.08 following stress/sleep intervention (z = −7.83, *P* < .001) on an 11-point Likert scale. Though changes in sleep duration for both groups were clinically meaningful, the stress/sleep intervention produced statistically larger improvements in sleep as compared to the diet/activity intervention (z = −3.79, *P* < .001). Sleep duration increased 26.39 minutes following diet/activity intervention (z = 3.16, *P* = 0.002) and 92.65 minutes following stress/sleep intervention (z = 5.912, *P* < 0.001).

**Conclusions:**

Findings suggest that diet and activity behavior change interventions can effectively improve outcomes within and between the behavior domains they directly target. Future research should identify common mechanistic pathways to inform development of interventions that efficiently change multiple health behaviors implicated in chronic disease morbidity and mortality.

## Introduction

Cardiovascular disease (CVD) affects approximately 48% of Americans [1] and accounts for over 900,000 deaths and $422.3 billion in annual direct and indirect medical costs [1]. The primary modifiable factors contributing to high prevalence and burden of CVD are lifestyle risk behaviors, such as diet, physical activity, sedentary behavior, and sleep. Behavioral risk factors tend to co-occur [2–4] and are prevalent in the US population: half of American adults exhibit two or more. Having multiple risk behaviors is associated with higher healthcare costs and heightened disease risk [2,5]. Moreover, the detrimental effects of risk behaviors are often synergistic, such that engaging in multiple risk behaviors increases the risk of chronic disease onset and mortality more than the additive effects of single risk behaviors [6–8]. To date, most research has focused on testing interventions on one risk factors at a time rather than seeking to address all relevant risk behaviors despite the fact that most Americans exhibit more two or more risks. Furthermore, little is known regarding what collateral risk improvements can be realized from a single intervention and what interventions confer the broadest range of benefits. To prevent CVD at the population level efficiently, effective and scalable lifestyle interventions are needed to change multiple risk behaviors.

Multiple health behavior change (MHBC) intervention (a single intervention that effectively improves multiple health risk behaviors) is a particularly efficient way to treat co-occurring CVD lifestyle risk behaviors. MHBC interventions are designed to take advantage of collateral effects that occur when a health behavior change that an individual makes in one domain leads to change in other health behaviors [9]. For example, Johnson and colleagues [10] reported that participants who showed improvement in one behavior (e.g., healthy eating, exercise) were 2.5–5 times more likely to make improvements in another health behavior. As supported in our prior work and consistent with a synergy hypothesis and goal systems theory [11], parallel improvements that occur across health behaviors may be attributable to motivation to achieve an overarching health related goal or a shared underlying behavior change mechanism as purported by behavior change theories such as social cognitive theory: e.g., an increase in confidence and self-efficacy may benefit both. Similarly, strategies learned to improve one behavior (e.g., goal setting, problem solving) may also be applicable to the other. Conversely, improvement may occur because increasing a health-promoting behavior substitutes for or reduces engagement in a health risk behavior [12]. What is currently unknown is which interventions can confer the greatest benefit across the most number of risk factors, a critical element to identify efficient pathways to treating the large proportion of the population with multiple risk factors.

In addition to physical activity and diet, a growing body of research suggests that stress and poor sleep also may convey disease risk [13–16]. For instance, Hoevenaar-Blom et al. [14] reported that short sleep duration was associated with a higher risk of coronary heart disease events. In response to this growing evidence in support of sleep duration as a key marker of CVD risk, the American Heart Association’s risk guidelines recently expanded to include the recommendation to achieve at least 7–9 hours sleep duration per night [17]. Additionally, research has shown associations between chronic psychological stress and cardiovascular disease morbidity [18] as well as between acute or discrete stressors [19] and cardiac events [20].

Like interventions for many health behaviors, current gold-standard interventions for stress and sleep target these factors singly and intensively, at high cost. For example, cognitive behavioral therapy for insomnia markedly improves insomnia, sleep duration, and sleep efficiency [21,22], yet treatment is generally delivered by specialized providers, limiting access and availability. Similarly, mindfulness-based stress reduction [23] and cognitive and behavioral therapies can improve stress management for healthy individuals [24] or those suffering from cardiometabolic disorders [25], but are also time- and resource-intensive for the health care system, the provider, and the patient. Packaging multiple evidence based interventions together could help individuals who have multiple behavioral risk factors, but engaging in more than one intensive or burdensome intervention is likely untenable for most individuals. Thus, understanding and harnessing synergistic and collateral behavior change strategies in MHBC interventionss might uncover feasible ways to preserve treatment effectiveness while improving efficiency.

On one hand, it may be necessary to expand existing MHBC interventions that target diet and activity behaviors to also include components that directly target stress management and sleep hygiene behaviors if the desired effect is to improve multiple risk factors at a time. However, adding such components and modules increases cost and burden, and potentially risks diminishing the potency of intervention components targeting diet and activity behaviors. On the other hand, it is possible that an existing MHBC intervention, such as one that only explicitly targets diet and activity risk behaviors may produce indirect or collateral improvements in stress and sleep. Indeed, MHBC interventions often produce such collateral improvements between distinct behavioral domains. For example, among participants who received MHBC intervention targeting both increased fruit and vegetable intake and reduced sedentary leisure team, collateral reductions in saturated fat consumption, even though saturated fat was not targeted [26]. Relatedly, there is associative evidence linking both healthy diet and regular physical activity with healthy sleep [27] and low perceived stress [28,29], However, no research to date has directly tested whether a MHBC intervention targeting diet and activity behaviors also produce collateral improvements in stress and sleep [22].

To assess whether an MHBC intervention targeting diet and activity risk behaviors produces collateral improvements in stress and sleep, we conducted a secondary analysis of the Make Better Choices 2 (MBC2) study [30]. The trial compared the effects on healthy diet and activity change of two different intervention conditions that directly targeted diet and physical activity (simultaneously or sequentially) versus an attention-matched comparison intervention that targeted improved stress and sleep. Both diet and physical activity interventions produced similar and sustained improvement on a composite diet and activity score, whereas the stress and sleep intervention did not [31]. The purpose of the present study was to compare the effects of the three interventions on stress and sleep to determine if, in addition to within-domain change, there were cross-domain effects on stress and sleep.

## Methods

Detailed methods, [30] and primary results of the trial along with the clinical trial protocol [31] are published elsewhere. Chicago area adults (n = 212) who displayed low MVPA, low fruit and vegetable intake, high saturated fat intake, and high sedentary leisure screen time were enrolled. MBC2 was a 9-month, 3-arm randomized control trial that compared effects on diet and activity risk behaviors of three different multicomponent interventions. Participants were recruited from July 18, 2012 until Jul 1, 2014 primarily through local flyers, transit advertisements, and newspaper advertisements. Potential participants were screened for eligibility criteria via an online questionnaire, phone screening call, in-person measures of blood pressure and HbA1c, and baseline recording of behaviors by smartphone app and accelerometer. All participants completed informed consent procedures and provided a signature on a written consent document. All three conditions incorporated self-monitoring and behavioral feedback via a custom smartphone application, psychoeducational materials related to the behaviors targeting for change, connected telephone-delivered health coaching, and small monetary incentives for attaining behavioral goals. The two diet and activity intervention conditions targeted improved fruit and vegetable intake, sedentary behavior and moderate to vigorous physical activity (MVPA) either simultaneously or sequentially. The third condition received an intervention that simultaneously targeted improved stress and sleep. Because the simultaneous and sequential diet and activity intervention arms had identical intervention components and functions and were found to have statistically comparable effects in the primary outcome trial, we treated them as one condition (diet/activity intervention) for this secondary analysis. The alternate intervention targeted reduction in stress, performance of relaxation exercises, and improvement in sleep. All study procedures were approved by the Northwestern University Institutional Review Board. The full study protocol is published as a supplement to the primary outcomes paper [31] and the study was registered on clinicaltrials.gov (NCT01249989)*.*

After providing written informed consent, participants were assigned using randomly permuted blocks with an allocation ratio of 2:2:1 to receive either the sequential or simultaneous diet and activity intervention, or the stress and sleep intervention. Participants were trained to use a custom smartphone application to self-monitor the behaviors targeted by their intervention throughout the 9-month study period. Participants assigned to diet/activity intervention used the custom-designed study app daily to self-monitor and receive feedback about dietary intake, sedentary leisure screen time, and MVPA. End goals for the first 12-weeks in the diet/activity conditions were 1)≤ 90 min per day of sedentary leisure screen time; 2) ≥5 servings of fruits and vegetables; and 3) ≥150 min per week MVPA. Participants assigned to the stress and sleep intervention were asked to self-monitor stress and sleep, and to engage in relaxation exercises daily. End goals for the first 12-weeks for the stress/sleep condition were 1) >7.5 hours of sleep per day and 2) 30% reduction in perceived stress. During the first 12 weeks of the intervention period, participant’s behavioral goals were tapered up as they worked to reduce the discrepancy between baseline and intervention goals. Diet and activity data were uploaded continually to a connected dashboard that coaches used to deliver tailored telephone counseling on a weekly (first 12 weeks), biweekly (weeks 13–24), or monthly schedule (weeks 25–39). Participants underwent 1-week assessment periods at baseline, and 13, 25, and 40 weeks. During assessment periods they self-monitored dietary intake, MVPA, sedentary leisure time, relaxation exercises, stress, and sleep daily without receiving feedback on any behaviors, regardless of randomized condition.

### Outcome measures

#### Stress.

Perceived stress was rated on an 11-point Likert scale that ranged from 0 (no stress) to 10 (highest stress). Single item stress assessments have shown good reliability and validity among children, adolescents, adults, and clinical populations [32–34]. At the start of each day, a pop-up window appearing in the study app displayed a dial that participants could modify to depict their stress level. They chose 3 times daily at which they were prompted to self-report stress. They were also able to use the app to adjust their stress ratings at any point throughout the day.

#### Sleep.

Each morning, participants used a smartphone questionnaire to report the duration of their last night’s sleep. They self-reported: (1) the time they went to bed, (2) the time they fell asleep, (3) the number of times they woke up during the night, (4) the total amount of time spent awake due to night awakening, (5) the time they woke up to start their day, and (6) the time they got out of bed. We computed sleep duration by subtracting minutes awake from total time in bed.

### Data analysis

Data for the purpose of this analysis were accessed on June 25, 2025. All study analyses were performed using Stata version 13 for Windows. Demographic variables were characterized using descriptive statistics. Each study outcome (daily stress and sleep) was evaluated as a function of time and intervention. The distribution of outcome variables was assessed for normality by computing a skewness value and visually inspecting histograms. Variables with a skewness score of >3 or whose histograms appeared to be abnormally distributed were transformed using a square root transformation. Models using the original, non-transformed variables were compared against those using the transformed counterparts.

Linear mixed models were conducted that compared the interventions’ respective effects on stress and sleep. Since linear mixed models can accommodate missing data across time, we used all available observations to maintain statistical power and avoid bias. The same modeling progressions and specifications were used for each outcome domain. Models included two levels: follow-up assessment observations were nested within persons. A random intercept was included for follow-up assessment period, and a person-level random intercept and slope were included for the time effect (i.e., assessment period). Models were evaluated with linear, quadratic, and cubic time effects (with time coded such that baseline was 0, 3-months was 1, 6-months was 2, and 9-months was 3). A fixed effect for intervention was included, coded such that the stress and sleep intervention (i.e., the comparison condition) was the reference group, and the combined diet/activity intervention was the comparator.

To evaluate whether each intervention differentially changed the outcome domain throughout treatment and follow-up, time X treatment interaction terms (3-time trends by 1 intervention effect) were also included. We used likelihood ratio tests to compare the models with each of the linear, quadratic, and cubic time terms interacted with condition, and interpreted the model if the inclusion of each respective interaction term was associated with statistically significant changes in model fit. Specifically, the higher order polynomial interactions with intervention were removed, in a backward manner, if found to be non-significant. Finally, post-estimation analyses were performed, and plots were created to probe significant intervention and time effects.

## Results

### Participant characteristics

Participants were primarily female (76%), non-white racial/ethnic identity (59%), and college educated (69%). Their mean age was 40.8 ± 11.9 years. Additional demographic details are reported elsewhere [31]. Table 1 reports the means and standard deviations of study outcome variables as a function of intervention and assessment time point.

**Table 1:** Means (+/- 95% CI) of Sleep Duration and Stress as a Function of Intervention Condition Across Time.

Intervention	Baseline	3 months	6 months	9 months
**Sleep Duration (Minutes)**				
**Sleep/Stress**				
N	44	39	34	30
Mean ± SD	412.47 ± 142.97	475.13 ± 77.95	492.94 ± 75.45	487.28 ± 88.86
**Diet/Activity**				
N	168	129	122	117
Mean ± SD	419.81 ± 110.44	412.76 ± 85.09	417.52 ± 79.85	426.08 ± 92.89
**Average Daily Stress**				
**Sleep/Stress**				
N	44	39	34	29
Mean ± SD	3.14 ± 1.83	0.9 ± 1.01	0.87 ± 1.08	1 ± 1.07
**Diet/Activity**				
N	168	136	126	119
Mean ± SD	3.51 ± 1.92	1.62 ± 1.64	1.71 ± 1.61	1.74 ± 1.53

The distribution of daily stress reports was approximately normal according to the histogram; the skewness value was < 3. Sleep duration, however, appeared to be slightly skewed, so a square root transformation was applied. Additional analysis was used to guide whether transformation should be used. We compared models using the original scores against those using the transformed equivalent and found no significant differences in fixed effect estimates and model fit [e.g., estimates that were statistically significant remained so and vice versa, and effect sizes were comparable]. Therefore, we elected to maintain the models using non-transformed variables to facilitate interpretation of results.

### Linear mixed models

**Fig 1:**
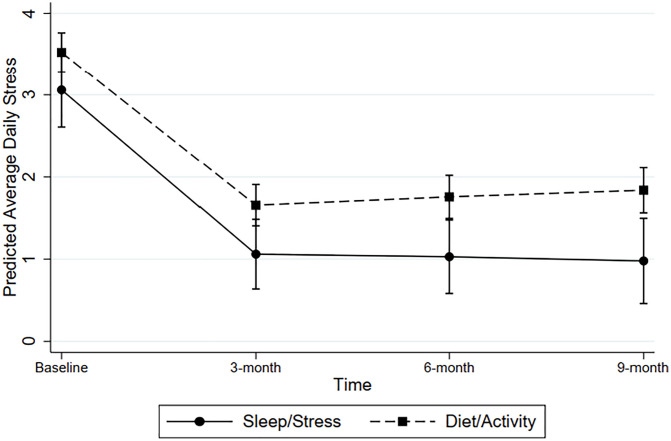
Predicted average daily stress (+/-95% CI) across time as a function of intervention type.

**Fig 2:**
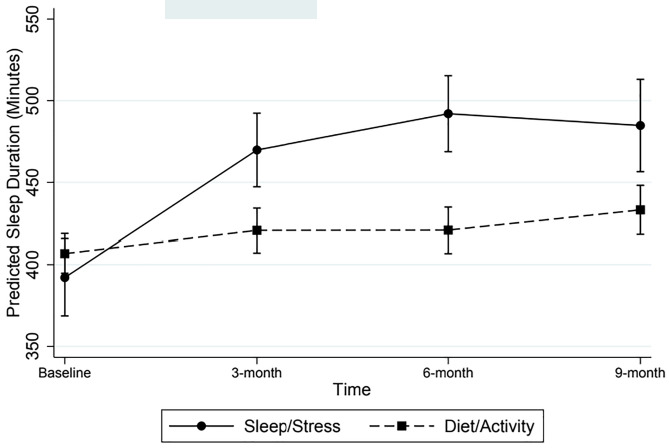
Predicted Mean (+/-95% CI) Sleep Duration Across Time as a Function of Intervention Type.

Fig 1 and Table 2 display the results of linear mixed models evaluating change in average daily stress across time. Removal of the cubic time by intervention interaction term [Likelihood ratio, χ^2^[1]=0.02, *P* = 0.88], and the quadratic interaction term [Likelihood ratio, χ^2^[1]=1.07, *P* = 0.30] did not significantly alter model fit. Therefore, only the linear time by intervention interaction was retained in the final model. Reductions in predicted average daily stress scores between baseline and 9 months were 2.08 for the stress and sleep intervention [z = −7.83, *P* < .01] and 1.68 for the combined diet/activity intervention [z = −12.25, *P* < .01] on an 11-point Likert scale. According to the final model (see Table 2), there were no significant differences between the time trend for the stress intervention versus the diet/activity and sleep intervention (z = 1.35, *P* = .18), suggesting that both interventions produced comparable improvements in stress. Models evaluating intervention effects on change in sleep duration are presented in Fig 2 and Table 2. Removal of the cubic time by intervention interaction term from the model did not significantly impact model fit (Likelihood ratio, χ^2^[1]=0.34, *P* = 0.56), but removal of the quadratic interaction terms did significantly reduce model fit (Likelihood ratio, χ^2^[1]=10.50, *P* = 0.01). Therefore, the quadratic time by intervention interaction term was retained in the final model.

**Table 2:** Summary of final linear mixed effects models predicting stress and sleep.

	Outcome Domain	
Fixed Effect Term	Average Daily Stress	Sleep Duration
Condition	0.46 [−0.04, 0.97]	14.61 [−11.69, 40.89]
Time	**−3.64 [−4.30, −2.99]**	**114.03 [64.83, 163.15]**
Time^2^	**1.98 [1.40, 2.55]**	**−40.71 [−77.82, −3.52]**
Time^3^	**−0.33 [−0.46, −0.20]**	4.33 [−3.66, 12.30]
**Linear**		
Condition X Time	0.13 [−0.06, 0.32]	**−84.61 [−122.54, −46.76]**
**Quadratic**		
Condition X Time^2^		**20.84 [8.28, 33.42]**

Note: Bolded values are statistically significant at the p < .05 level.

Values corresponding to each fixed effect variable are the coefficient with brackets representing 95% confidence intervals.

There were clinically meaningful and positive changes in sleep for both intervention groups, but the stress and sleep intervention resulted in statistically larger improvements in sleep as compared to the diet/activity intervention (z = −3.79, *P* < .01). For the stress and sleep intervention, estimated improvement in sleep duration followed a quadratic trend, such that improvement was greatest from baseline to 3-months (z = 4.56, *P* < 0.01), and then continued to improve less rapidly from 3-months onward (z = −2.15, *P* = 0.03). The diet/activity intervention produced a different trajectory of change in sleep duration, such that there was substantially less initial improvement (z = −4.39, *P < 0.01*), but a gradual increase in rate of improvement (relative to the stress/sleep intervention) at later timepoints. Overall, the stress and sleep intervention produced the larger improvement in sleep duration, yielding a 92.65-minute increase in estimated sleep duration from baseline to 9-months (z = 5.91, *P* < 0.01). The diet/activity intervention also produced a significant but smaller improvement in sleep, increasing estimated sleep duration by 26.39 minutes (z = 3.16, *P* = 0.01).

## Discussion

The present study investigated whether behavioral interventions targeting diet and physical activity also incidentally improved stress and sleep, and whether these improvements were comparable in magnitude to those produced by an intervention that was designed to directly target stress and sleep. In our sample, both the stress/sleep and diet/activity interventions significantly reduced stress to a similar degree. Both interventions also significantly improved sleep duration, although the magnitude of improvement between baseline and 9 months was greater for those treated with the stress and sleep intervention.

Consistent with goal systems theory, participants were likely drawn to participate in the study due to an overarching desire and motivation to engage in a process of changing health behaviors [35]. Thus, despite being focused on a particular subgoal per the study condition to which a participant was randomized, other behavior changes may have resulted via goal facilitation and conflict avoidance. For example, if the overarching goal was to be healthier and the subgoal was to be more physically active, an individual might also use physical activity for stress reduction in an effort to serve the overarching goal. To avoid goal conflict, one might go to bed early rather than be tempted by eating late night snacks. Our results support the hypothesis that collateral improvement in stress and sleep can be produced by behavioral interventions that target and effectively improve diet and activity health risk behaviors.

To our knowledge, this is the first study to suggest that a MHBC intervention targeting

diet and physical activity can also foster collateral improvements in stress and, to a lesser degree, in sleep, even when neither stress nor sleep is directly addressed by intervention content. These findings align with prior results demonstrating that targeting a health risk behavior can also produce accompanying “tag along” improvements in untargeted health risk behaviors [26,31].

It is informative to contextualize the effects of the present intervention on stress and sleep relative to the effects seen by interventions designed to explicitly target these domains. In the present study, diet/activity intervention produced improvements in stress that were comparable in magnitude to those produced by interventions that were designed explicitly to alleviate stress [36]. Moreover, although diet/activity intervention produced a smaller improvement in sleep than the stress and sleep intervention, the 26.39 minute increase in sleep duration that diet/activity intervention did produce was statistically significant and was maintained through 9 months. This change in sleep duration was enough to move the average from just below to within the recommended 7–9 hour range and is of similar magnitude to improvements observed as a result of some gold-standard clinical interventions that directly target sleep [11,37,38]. Thus, in the context of a population with behavioral risk factors and with sleep disturbance that meets subclinical rather than clinically significant criteria for disorder, such an approach could be appropriate.

The findings of the current study have important implications for efficiently addressing chronic disease at the population level. Our results suggest that effective diet and activity behavior change interventions might offer cross-domain improvements in stress and sleep without incurring the added burden and cost needed to directly target those domains. Additionally, as compared to in-person treatment for stress or sleep, remotely delivered, connected, technology-assisted MHBC coaching offers high scalability and reduced intervention costs [34,36,39]. Altogether, the meaningful improvements in stress and sleep produced by our active MBC2 intervention suggest that health promotion targeting diet and activity improvement alone may be an appropriate low-burden, first-line intervention for patients who present with multiple health risk behaviors.

Strengths of the study include a racially diverse sample and an internally valid clinical trial design, including strong treatment fidelity [30,31]. Limitations include the potential for self-report bias, since the study did not include objective biomarker assessments that would now be feasible. Additionally, although study entry criteria required that participants exhibit all four diet and physical activity risk behaviors, they did not require that participants report a threshold of high stress or insufficient sleep. However, study recruitment material did state that participants might be randomized to a lifestyle intervention that addressed stress and poor sleep. Presumably, therefore, the trial attracted individuals who perceived a benefit from reducing stress or improving sleep. Notably, groups had moderate levels of stress, were below or just within the lowest recommended sleep duration at baseline.

## Conclusion

Being able to reduce multiple cardiovascular risk behaviors simultaneously is inherently more efficient than needing to reduce them separately, one at a time. Results of this secondary analysis suggest that intervening to improve risky diet and physical activity behaviors may produce positive effects on other outcomes such as stress and sleep. Research that can uncover cross-domain positive benefit could help meet broader population needs, particularly given the high co-occurrence of insufficient sleep, high stress, unhealthy eating, excessive screen time, and low MVPA.
